# Individualized Treatment of Genotype 1 Naïve Patients: An Italian Multicenter Field Practice Experience

**DOI:** 10.1371/journal.pone.0110284

**Published:** 2014-10-23

**Authors:** Alessandra Mangia, Giovanni Cenderello, Alessandra Orlandini, Valeria Piazzolla, Antonio Picciotto, Massimo Zuin, Alessia Ciancio, Giuseppina Brancaccio, Paolo Forte, Vito Carretta, Anna Linda Zignego, Nicola Minerva, Gaetano Brindicci, Massimo Marignani, Gianluca Svegliati Baroni, Gaetano Bertino, Giuseppe Cuccorese, Leonardo Mottola, Maria Ripoli, Mario Pirisi

**Affiliations:** 1 Hospital IRCCS “Casa Sollievo della Sofferenza”, Liver Unit, San Giovanni Rotondo, Italy; 2 Galliera Hospital, Infectious Diseases, Genova, Italy; 3 Hospital of Parma, Unit of Infectious Diseases and Hepatology, Parma, Italy; 4 University of Genova, Department of Internal Medicine, Gastroenterology Unit, Genova, Italy; 5 “S. Paolo” Hospital, University of Milan, Department of Medicine, Milan, Italy; 6 AOU “San Giovanni Battista” - Molinette, Department of Medical Sciences, Torino, Italy; 7 Second University of Naples, Department of Infectious Diseases, Naples, Italy; 8 “G. Careggi” Hospital, Department of Gastroenterology, Florence, Italy; 9 Hospital of Venosa, Unit of Medicine, Venosa, Italy; 10 University of Milan, Department of Experimental and Clinical Medicine, Milan, Italy; 11 Hospital of Canosa, Department of Internal Medicine, Canosa, Italy; 12 University of Bari, Clinic of Infectious Diseases, Bari, Italy; 13 “S. Andrea” Hospital, Department of Gastroenterology, Rome, Italy; 14 University of Ancona, Department of Gastroenterology, Ancona, Italy; 15 “Vittorio Emanuele” Hospital, Liver Unit, Catania, Italy; 16 “R. Dimiccoli” Hospital, Department of Gaastroenterology, Barletta, Italy; 17 Università degli Studi del Piemonte Orientale, Department of Internal Medicine, Novara, Italy; University of Catania, Italy

## Abstract

**Background:**

Triple therapy including Telaprevir or Boceprevir still represents in many European countries the standard of care for patients with Hepatitis C Virus genotype 1 infection. The number of patients who received this treatment resulted generally lower than expected. We investigated, among naïve patients, number and characteristics of treatment candidates who were started on triple or dual therapy in comparison to those who were deferred.

**Patients and Methods:**

621 naïve treatment candidates were prospectively evaluated at each center. Factors associated with decision to defer or treat with dual or triple therapy were investigated by univariate and multivariate analyses. Rates of Sustained Virological Response and safety profile were analysed.

**Results:**

Of candidates to treatment, 33% did not received it. It was mostly due to high risk of Interferon-induced decompensation. Of 397 patients who were started on treatment, 266 (67%) received triple, 131 dual. Among patient receiving treatment, unfavorable IL28B, severe liver damage and higher albumin were independently associated with the physician decision to administer triple therapy. Sustained Virological Response after dual therapy was 66.4%, after triple 73.7% (p = 0.14). 142 patients received Telaprevir. The choice of Telaprevir-based therapy was associated with higher Body Mass Index and advanced liver disease. Sustained Virological Response rates were 71.1% after Telaprevir and 76.6% after Boceprevir.

**Conclusions:**

Individualizing treatment with available regimens allows to maximize Sustained Virological Response and to reduce the number of patients who remain untreated. High proportion of patients with severe liver damage urgently need Interferon free treatment.

## Introduction

Worldwide HCV infection affects more than 180 million people [1]. In Italy, it is estimated that more than 1.4 million of people carry the virus [2]. However, no more than 20% of patients with advanced liver disease receive treatment [3]. Despite anticipated esteems of high numbers of candidates, patients treated with triple therapy (TT) including Telaprevir (TVR) or Boceprevir (BOC), yet representing the standard of care for HCV genotype 1 in many European countries, ranges from 44% to 49% of the expected numbers [4], [5]. In US, Chen et al showed that the rate of subjects initiating TT (18.7%) was nearly identical to the treatment rate reported with dual therapy (DT) [6]. In Europe, in a single center study, half of treatment candidates were not started because of safety concerns [5]. Both EU and US studies refer to a mixed population of prior treatment failures and naïve patients [4]–[6].

The safety profile of the TT combination appeared poor. Hospitalization during the first 12 weeks of treatment were frequent with anemia being responsible for them in 65% of cases [5], [7]. In the CUPIC cohort focusing on patients with very advanced liver disease, severe anemia was reported in 13% of patients on TVR and in 9% of those on BOC. In the same cohort, rash was associated with treatment discontinuation in 5.3% of patients receiving TVR [8]. However, the occurrence of side effects seems to be lower in previously untreated patients, as anemia rates <8.5 g/dL were registered in 5–9% in the SPRINT-2 study and in 4% in the ADVANCE [9], [10] in comparison to 14% in RESPOND and REALIZE [11], [12].

Beside of side effects, other factors limited the proportion of patients receiving triple combination treatment. Indeed, candidacy to TT was largely debated at a country level due to the complexity of the regimens. In Italy, additional reasons for barrier to treatment were represented by treating centers selection: only some centers were allowed to perform TT on the basis of predefined skills and on the availability of specific tools that include a quick turn-around for HCV RNA assays results, IL28B genetic testing and availability of transient elastometry. Moreover, Italian Guidelines advised treatment of naïve or treatment experienced patients with advanced fibrosis and cirrhosis, but at a local level some regions decided to select for TT regimens only patients with a prior treatment failure [13]. Finally, it was suggested that a proportion of patients with favorable baseline factors may continue to be treated with dual therapy to spare economical resources in consideration of the higher costs of DAA [14].

At this stage it remains unclear what proportion of the total HCV genotype 1 naïve patients eligible to triple therapy took advantage of TT in the real world. With the more convenient safety profile of the coming interferon free regimens it may be interesting to clarify whether reasons not to initiate therapy are related to the presence of a mild disease or to poor chances of achieving SVR due to high risk of side effects or because of coexistence of unfavorable baseline predictors [15], [16]. The main goal of this analysis is to prospectively evaluate physicians preferences on treatment decision in our country, in naïve genotype 1 patients followed at different centers. Secondary objectives were to assess the virologic response to TT in naïve patients, in a real world experience.

## Patients and Methods

This study was a non interventional prospective nationwide multicenter cohort study conducted at 22 Italian centers since June 2012, when the genetic samples of candidate naïve patients with HCV genotype 1 infection were centralized and tested. Patients with decompensated cirrhosis in Child-Pugh class ≥B7 were not included. Screening started on January and enrollment on February 2013 when TT become available in Italy. Only patients who completed 12 week of follow up by May 2014 are included in this analysis. Patients with history of previous treatments, as well as patients with HIV or HBV co-infection, were not eligible. Diagnosis of cirrhosis was made by liver biopsy or by non invasive test, transient elastometry or APRI. Written informed consent was obtained for the participation in the perspective study as a whole. The protocol was conducted in accordance with the Declaration of Helsinki and was approved by the coordinating center's Ethic Committee (Independent Ethic Committee –IEC–, IRCCS “Casa Sollievo della Sofferenza”).

As the aim of the study was to obtain a picture of physician behavior in the real life, treatment or deferral decision were made individually by the physician in charge and were not influenced by a common protocol. All naïve patients consecutively observed were included. TT with both first generation protease inhibitors (PI), TVR or BOC was allowed. Patients were monitored according to physician preference but a minimum of twice a month visit and laboratory evaluation was performed at each center.

Anemia was graded as severe when Hb levels were 9 g/L, neutropenia when neutrophil count lower than 800 cells/mm^3^ was registered. Treatment was discontinued when Hb levels were <8.0 g/L without improvement after ribavirin dose reduction and blood transfusion. Granulocyte colony stimulating factors were not admitted and neutropenia was managed by PegInterferon dose reduction.

### Treatment

DT and TT were prescribed in accordance with the National guidelines and stopping rules [13]. For TT, response guided therapy was adopted in non cirrhotic patients, while cirrhotic received 48 weeks of treatment.

### HCV RNA monitoring

HCV RNA levels were measured at baseline and at weeks 4, 8, 12, 16, 24, 36, and 48 during treatment, and 12 and 24 weeks off treatment, by a real-time PCR based assay, either COBAS AmpliPrep/COBAS TaqMan (Roche Molecular Systems, Pleasanton, California) with a lower limit of detection of 15 IU/ml, or m2000 SP/m2000 RT (Abbott Molecular, Des Moines, Illinois), with a lower limit of detection of 12 IU/ml. In this analysis, virological responses at week 12 after treatment were evaluated. Non cirrhotic patients with eRVR defined as undetectable HCV RNA result at week 4 and 24 in TVR arm and patients undetectable at week 8 and 24 on BOC arm received a course of treatment of 24 weeks only. Stopping rules were used in accordance with Italian guidelines [13].

### IL28B genotype

IL28B genotyping (rs1297860) was centralized (Liver Unit, IRCCS San Giovanni Rotondo). Genotype was determined for all patients candidate to treatment. Patients DNA were extracted from peripheral blood using standard methods. Genotyping was performed using TaqMan allelic discrimination assay, as described [17]. Hardy-Weinberg's equilibrium of the IL28B polymorphism was tested for the study population.

### Fibrosis assessment

Fibrosis staging was defined by liver histology according to Scheuer's classification in 36% of subjects [18]. All the patients received a non invasive evaluation including transient elastometry using the threshold of 12.5 KPa to define cirrhosis or biomarkers. The biomarker used was APRI. The threshold used to define cirrhosis by APRI was ≥2.

### Statistical analysis

Data are presented as percentages for categorical variables and mean with standard deviations for continuous variables. Univariate analyses of baseline or pretreatment variables were performed by two-sided t test and chi squared with Fisher's exact test when appropriate. Within-group, comparisons were made using the Wilcoxon test. Baseline variables with P values ≤0.05 by univariate analyses entered into multivariate logistic regression model to find independent factors, which were expressed by Odds ratio (OR) with 95% confidence intervals (95% CI) for predictors of different of physician decisions or treatment responses. Backward elimination procedure was used. Statistical analysis was performed by SPSS version 10.0 (SPSS, Chicago IL). Median values of quantitative variables were compared using a nonparametric test (Mann-Withney two-tailed test).

Efficacy analyses were performed on an intent-to-treat basis. Missing virological measurements were imputed as treatment failures.

## Results

### Patient characteristics

**Figure 1:**
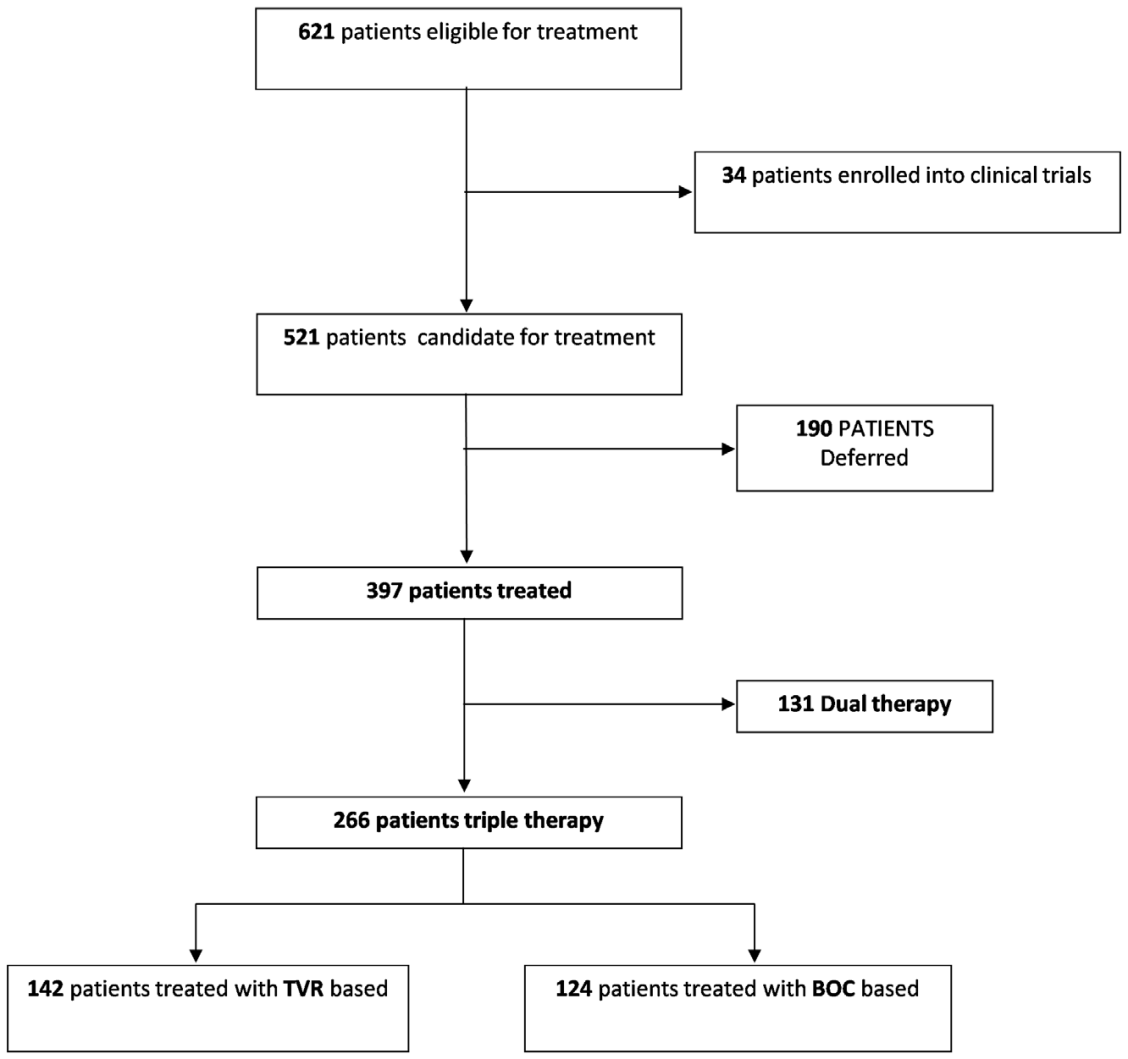
Patient disposition in the study.

Among 621 consecutive patients with chronic HCV genotype 1 infection who were referred to the 22 outpatients clinics involved in this study, we restricted the analysis to the 587 patients who did not enter clinical trials ongoing in the same period of time at 4 out of 22 participating centers (Fig. 1). Baseline characteristics of patients overall and by treatment or deferral decision are shown in Table 1.

**Table 1:** Characteristics of naïve candidate patients by physician decision to treat or not.

Characteristics	Pts treated N = 397 (67.6)	Pts deferred N = 190 (32.4)	P Value
Male, no (%)	246 (62.0)	97 (51.1)	0.01
Mean age ±SD (yrs)	54.0±12.7	53.1±14.3	0.44
Mean BMI±SD (Kg/m^2^)	24.7±9.0	23.8±9.5	0.10
HCV genotype, no (%)			
1	50 (12.5)	19 (10.0)	
1a	100 (25.2)	46 (24.2)	0.68
1b	247 (62.3)	125 (65.8)	
Mean baseline HCV RNA x 10^6^ IU/mL±SD	3.0±1.2	2.0±2.9	0.26
Mean ALT IU/L±SD	87.5±66.3	69.0±55.8	0.001
Mean platelet count x 10^3^/µL±SD	180±68	202±73	0.0001
rs12979860 genotype frequency*			
IL28B carriers CC, no (%)	95 (27.1)	36 (18.9)	
IL28B carriers CT, no (%)	196 (53.1)	121 (63.7)	0.12
IL28B carriers TT, no (%)	60 (16.9)	33 (17.4)	
Cirrhosis (≥12.5 KPa) no (%)	110 (27.8)	41 (21.5)	0.14
APRI score ≥2^§^±SD	120 (32.1)	80 (41.9)	0.02
Baseline Hb g/dL±SD	13.9±8.1	12.8±4.3	0.06
Baseline Albumin g/dL±SD	3.9±0.9	3.4±1.5	0.0001

*IL28B rs12979860 undetermined in 47 cases among treated patients; ^§^ not available in 22 cases among treated patients.

### Patients treated versus patients deferred

Overall 397 (67%) naïve patients aged >18 were treated while the remaining 190 were not (Fig. 1). Therefore, in our country, a consistent proportion of patients with HCV genotype 1 evaluated for treatment did not receive any of the currently available therapies. Of patients who were started on therapy, 62% were male, while a higher proportion of female was observed among untreated patients. A favorable IL28B profile was observed at comparable frequency between treated and untreated patients. Among treatment candidates, genotype 1b was identified in 62%, genotype 1a, in 25%. In the remaining, subtypes were undetermined. HCV RNA levels were not associated with the decision of treating or not. Among untreated patients, rates of ALT were lower. Overall, advanced fibrosis or cirrhosis, as defined by elastometry, was present in 151 subjects (38.0%) undergoing treatment. The proportion of patients with cirrhosis was higher in treated than in untreated (21.5%). This evidence suggests that physician's decision to treat or not was mostly driven by reasons related to the urgency of treatment determined by an advanced liver damage. However, this liver damage had to be not at risk of decompensation because when baseline Hb, albumin levels and PLT counts were investigated, as shown in Table 1, low PLT counts and albumin levels were significantly associated with decision not to treat (p = 0.001 and p = 0.0001, respectively). Consistently, APRI score ≥2 was observed at significantly higher proportion in patients untreated as compared to treated (41.9% vs 32.1%). These findings suggest that evidence of advanced liver damage associated with low risk of decompensation rather than favorable predictors of response oriented physician choices. Independent predictors of treatment resulted higher albumin levels OR = 0.79; 95% CI 0.67–0.93 (p = 0.005) and IL28BCC OR = 0.54; 95% CI 0.34–0.83 (p = 0.01).

### Characteristics of patients receiving treatment: dual versus triple therapy

Of 397 patients who were treated, 266 (67.0%) received TT, while the remaining 131 initiated dual therapy (Fig. 1). Baseline factors associated with the choice of DT or TT are reported in Table 2. Of patients who started TT, the proportion of male, was comparable with that of subjects candidate to DT. Mean age of patients initiating TT was higher than mean age of patients initiating DT (p = 0.02). In the group of TT, 34.6% of patients had diagnosis of cirrhosis, this percentage was significantly higher than the corresponding 13.6% rate observed in patients receiving DT (p = 0.0001). Mean PLT count was lower among patients receiving TT than among those receiving DT (p = 0.0001). No difference in HCV subtype distribution was observed. In addition to older age, severe liver disease, proven also by baseline PLT counts, IL28BCC genotype was differently distributed between the two treatment groups. Indeed, we observed an association between CC and dual therapy (p = 0.0001). Higher albumin levels were observed among patients receiving TT as compared to DT (p = 0.0001).

**Table 2:** Characteristics of patients treated with triple or dual combination.

Characteristics	Pts receiving triple Tx	Pts receiving dual Tx	P Value
	N = 266 (67.0)	N = 131 (33.0)	
Male, no (%)	169 (63.5)	75 (57.3)	0.27
Mean age ±SD (yrs)	55.1±12.5	51.9±13.0	0.021
Mean BMI ±SD (kg/m^2^)	35.8±15.6	22.8±31.1	0.46
HCV genotype, no (%)			
1	26 (9.9)	23 (17.6)	
1a	75 (28.6)	24 (18.3)	0.021
1b	164 (61.5)	83 (63.4)	
Mean baseline HCV RNA x 10^6^ IU/mL±SD	3.6±1.6	2.3±3.1	0.34
Mean ALT IU/L±SD	84.7±3.8	89.3±6.5	0.52
Mean platelet count X10^3^/µL±SD	162±76	203±76	0.0001
rs12979860 genotype frequency*			
IL28B carriers CC, no (%)	49 (24.0)	46 (36.8)	
IL28B carriers CT, no (%)	135 (57.8)	61 (48.8)	0.016
IL28B carriers TT, no (%)	42 (18.2)	18 (14.4)	
Cirrhosis (**≥**12.5 KPa), no (%)	92 (34.6)	18 (13.6)	0.0001
APRI score ≥2§±SD	77 (31.6)	43 (32.8)	0.89
Baseline Hb g/dL±SD	13.4±4.6	14.9±12.3	0.07
Baseline Albumin g/dL±SD	4.1±0.7	3.6±0.1	0.0001
SVR, no (%)	197 (74.0)	87 (66.4)	0.14

*IL28B rs12979860 undetermined in 41 and 6 patients treated with TT or DT, respectively;

§ not available in 22 cases among patients receiving TT.

**Figure 2:**
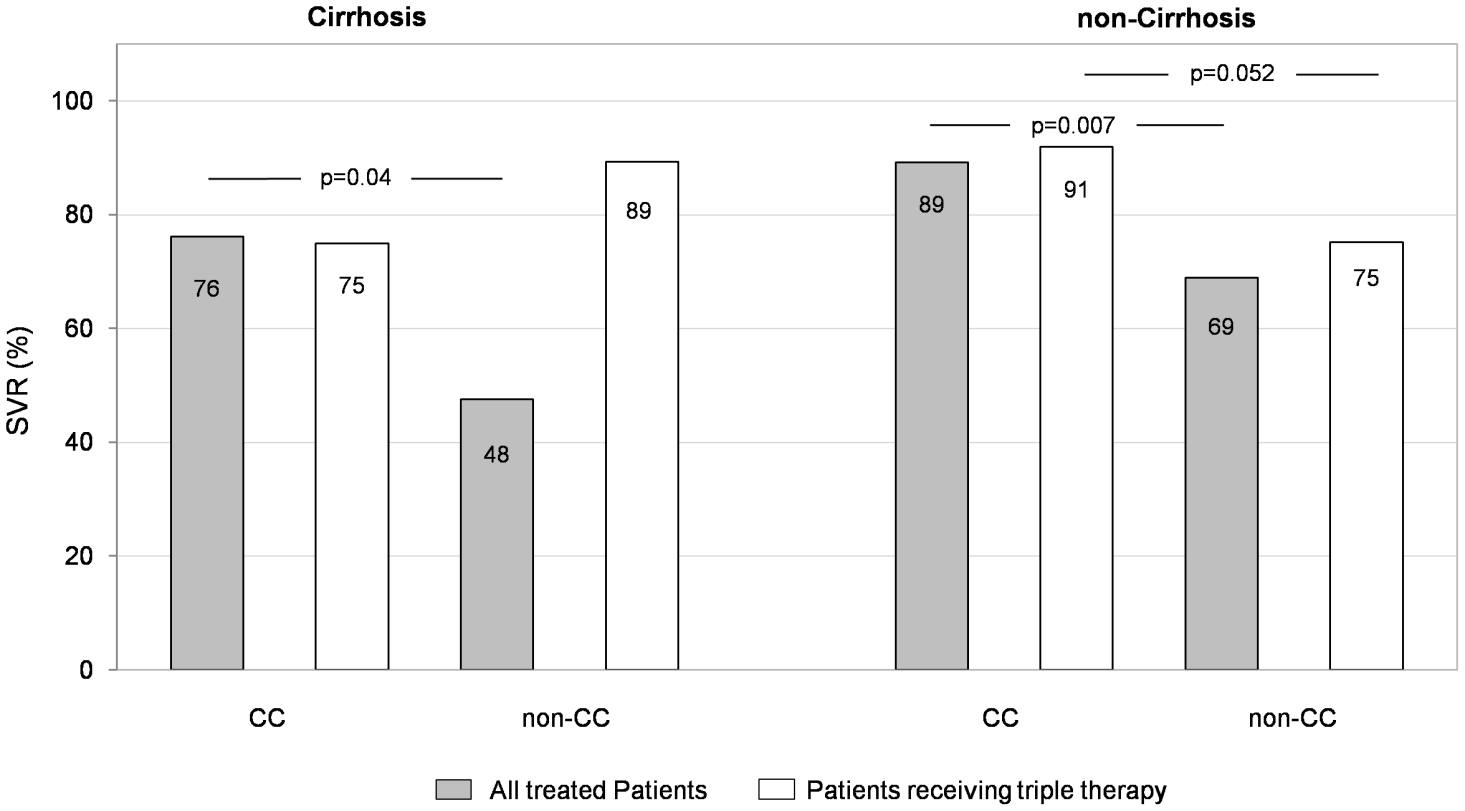
Association between SVR and IL28B genotype. All treated patients (gray) or patients receiving triple therapy (white) were analysed by cirrhosis status and IL28 CC or non CC.

As shown in Fig. 2 in patients with IL28B non-CC and cirrhosis, the addition of PI increased SVR rates.

By multivariate analysis, independent predictors associated with the choice of triple therapy were severe liver damage OR = 1.4, 95% CI 1.06–1.86 (p = 0.018), IL28B non-CC OR = 2.45, 95% CI 1.35–4.46, (p = 0.004) and higher albumin levels OR = 1.89, 95% CI 1.28–2.80 (p = 0.001).

### Efficacy of therapy: dual versus triple therapy

In intent-to-treat analysis, among the 131 naïve patients who received DT, HCV RNA was undetectable at 12 weeks of follow up in 87 (66.4%) (95% CI: 58.3–74.5). The corresponding rate among 266 naïve patients receiving TT was 74.0% (95% CI: 68.7–79.3). Eight and 5 patients experienced a relapse with DT therapy or TT, respectively (p = 0.16). Of interest, in 33 of 174 patients without cirrhosis who received TT it was possible to administer a short course of TT in accordance with eRVR. Relapse was registered in 12.5% of patients. Factors independently associated with SVR to TT were investigated by uni and multivariate analysis including TVR or BOC as covariate. Higher proportion of patients with advanced liver damage was registered among non responders as compared to responder patient 47.1% vs 25.5% (p = 0.001). IL28BCC was observed in 28% of responders as compared to 11.6% of non responders (p = 0.008) (Fig. 2). Multivariate analysis confirmed IL28BCC as the independent predictor OR = 0.34, 95% CI 0.14–0.83 (p = 0.018).

Discontinuation rate was lower than reported in other real life studies, as 23 of 266 patient on TT (8.7%) and 7 of 131 on DT (5.3%) discontinued due to side effects. Among patients who were treated with TT, all the discontinuations were due to side effects, while among patients receiving DT, only 2 of 6 discontinued due to side effects. The rate of patients developing anemia during treatment was 22.2% for DT and 39% for TT (p = 0.24). Only 5% on DT versus 21% of patients on TT required blood transfusion (p = 0.26). Neutropenia was registered in 11.1% of patients on DT and in 22% of patients on TT (p = 0.46). No cutaneous rash was observed among patients on DT, the corresponding rate was 14.2% among patients receiving TT (p = 0.15).

Of patients receiving TT, 13 discontinued TVR and 9 discontinued BOC; it was due to adverse events, represented by anemia in 2 cases receiving TVR, rash/pruritus of moderate grade in 4, and Dress syndrome in 1. Severe neutropenia and thrombocytopenia were associated with the remaining patients. For patients receiving BOC, 6 cases of pneumonia required treatment discontinuation and hospitalization, 1 patient had severe neutropenia. Other reasons for treatment discontinuation included generic intolerance.

### Characteristics of patients receiving TVR or BOC based triple therapy

Of 266 patients who received triple therapy, 142 (53.3%) were treated with TVR and 124 (46.7%) with BOC-based combination. In order to understand reasons for physicians preferences, baseline characteristics of patients enrolled to BOC were compared with those of patients enrolled to TVR (Table 3). As shown, in the latter group significantly higher number of patients had higher mean BMI (25.9 vs 22.8, p = 0.001) and low PLT count (143±71 versus 182±75, p = 0.0001). By contrast, in the former group, higher proportion of patients had less advanced liver damage (p = 0.0001). No differences were observed in the distribution of HCV subtypes, IL28B genotypes, APRI score and albumin, between the two treatments. Notably, no difference in the rate of treatment discontinuation by different PI were registered. At multivariate analysis, the factors independently associated with physician preferences for TVR resulted higher BMI (OR = 0.91; 95% CI 0.87– 0.95 p = 0.001), cirrhosis (OR = 0.66; 95% CI 0.48–0.91 p = 0.013) and PLT count (OR = 1.01; 95% CI 1.00–1.02 p = 0.001).

**Table 3:** Baseline factors orienting physician choices FOR TVR or BOC.

Characteristics	Pts receiving TVR	Pts receiving BOC	P Value
	N = 142 (53.3)	N = 124 (46.7)	
Male, no (%)	98 (69.0)	75 (60.5)	0.22
Mean age ±SD (yrs)	55.6±10.7	54.4±14.3	0.44
Mean BMI±SD (kg/m^2^)	25.9±6.4	22.8±8.5	0.001
HCV genotype, no (%)			
1	16 (11.4)	10 (8.1)	
1a	42 (29.3)	34 (27.1)	0.71
1b	84 (59.3)	83 (63.4)	
HCV RNA x 10^6^ IU/mL±SD	4.4±2.0	2.3±3.0	0.26
Mean ALT IU/L±SD	92.0±62.9	80.1±60.4	0.13
Mean platelet count x 10^3^/µL±SD	143±71	182±75	0.0001
rs12979860 genotype frequency*			
IL28B carriers CC, no (%)	23 (22.3)	26 (24.2)	
IL28B carriers CT, no (%)	64 (62.1)	71 (54.8)	0.67
IL28B carriers TT, no (%)	16 (15.6)	26 (21.0)	
Cirrhosis (**≥**12.5 KPa) no (%)	66 (46.5)	26 (21.0)	0.0001
APRI score ≥2^§^±SD	42 (32.5)	35 (30.4)	0.82
Baseline Hb g/dL±SD	13.2±5.0	13.7±3.9	0.91
Albumin g/dL±SD	4.1±0.4	4.0±0.6	0.46

*IL28B rs12979860 undetermined in 40 patients treated with TVR and 1 treated with BOC; ^§^not available in 13 cases among patients treated with TVR and in 9 patients treated with BOC

Head to head comparison of the two treatment regimens was not aim of this study, nevertheless, we observed that rates of SVR between triple therapies including BOC or TVR are comparable (71% vs 77%; 95% CI: 63–79 and 70–84, respectively). To understand whether unfavorable baseline factors identified according to physician's preferences in each treatment might have impaired rates of SVR in TVR group, we performed adjustments for cirrhosis and BMI in a further analysis of predictors of SVR, considering TVR as the selection variable. BMI and cirrhosis were not independent predictors of SVR.

## Discussion

The recently released European guidelines for the treatment of hepatitis C state that, when newer therapy options are not available, a first generation PI in combination with Peg-IFN and RBV represents the first option for treatment of genotype 1 infected patients [14]. Given the diversity of European population and reimbursement practices, these recommendations differ from those released in US that did not recommend the use of first generation PI [19]. Until recently, TVR and BOC were evaluated mostly in clinical trials. Data on efficacy and safety in real life are derived from the CUPIC study that was performed to explore applicability of this combination in previously treated patients with very advanced liver disease [8] and from the large German PAN cohort whose SVR is not yet available [20]. This is a “real world” multicenter non interventional study, representative of Italian physician behavior and treatment decision in newly diagnosed patients. The study focus on patients seeking treatment who were firstly seen at 22 different Italian centers. Our results suggest that in naïve patients Italian physicians decided to defer treatment in 32% of cases. This rate would have been much higher if instead of not treating Italian physician would not have used the standard dual combination in 22% of treatment candidates. As a consequence, rather than 54%, only 32% of patients remained untreated. Of patients who were not treated, no more than 60% had mild or moderate fibrosis and significantly higher PLT count suggesting evidence of a mild disease. As patients with Child-Pugh ≥B7 were excluded from this study, involving only previously untreated patients, we assumed that reasons not to treat would have been an initial disease in the vast majority of cases, yet 15 of 37 patients with PLT count below 100.000/µL and albumin <3.5 mg/dl (40%) remained untreated, among naïve patients. For these patients IFN free options are urgently needed [21], [22].

Selecting for Peg-IFN and ribavirin therapy, 22% genotype 1 naïve patients with favorable baseline characteristics resulted in a more effective strategy than allowing liver disease to progress without treatment in waiting to have access to new DAA. In this respect, our results partially differ from those attained a few months ago in another real life study where 60% of patients candidate to TT were treatment experienced [5]. In that study, higher proportion of patients remained untreated. The decision to defer treatment was based on treatment related safety concerns in 64% of cases and on patients preferences in 32%. In this study of 151 patients with cirrhosis, 60% received TT, 23% remained untreated and 12% received DT. The decision not to treat was based on safety in 50% of cases, while, due to the different characteristics of the studies, in the remaining cases it was dependent on the presence of mild liver damage.

The need of adding a third drug to Peg-IFN and RBV, in real life, appears challenging in patients with baseline unfavorable characteristics. Indeed, among 266 patients who received TT, the final decision to start this regimen instead of DT was supported by two independent predictors: an unfavorable IL28B profile and an advanced liver damage in the absence of risk of decompensation. As a consequence of this selection for treatment candidates to DT over TT, 66% rates of SVR registered after DT were higher than those traditionally reported in genotype 1 after Peg-IFN and ribavirin [23], [24]. On the other hand, despite the unfavorable IL28B profile, considering that patients with high risk of decompensation were excluded, among patients receiving TT SVR rates registered in this study are absolutely comparable to the response rates reported in registration trials on TT, in naïve patients [9], [10]. When compared to other real life studies not focusing on CUPIC-like patient population [25], rates of SVR in our study were similar. A 63.4% SVR response rate was observed in PAN cohort including 273 naïve patients receiving TVR [20]. However, missing information on liver disease assessment from that study prevent any comparison. In the same cohort, 85 naïve patients receiving BOC achieved on treatment response of 71.6% at week 12. In our study SVR rate for patients receiving BOC was comparable with this on treatment rate, although since no SVR results are currently available from that cohort, conclusions cannot be driven.

In this study, patients candidate to TVR based regimen had higher BMI and more advanced liver damage. Of course this is not a randomized controlled study and selection bias cannot certainly be ruled out. As the study was not aimed to an head to head comparison, we cannot reach conclusions on the different efficacy of the different TT combination.

As high incidence of side effects has been so far demonstrated using first generation PI, mostly in patients who were treatment experienced, we investigated how this treatment was tolerated in naïve patients, in real life. We observed that, carefully monitoring patients every 4 weeks during the initial 12 week treatment period, the risk of discontinuation is low and comparable to that of patients receiving DT. Indeed, the rate of discontinuation in patients receiving TT in this study was relatively low, although in every patient, treatment withdrawal was due to side effects in particular to severe anemia, thrombocytopenia or pulmonary infections. These findings are in keeping with other studies suggesting for example that the proportion of patients requiring hospitalization during the first 12 weeks of TT was significantly lower among naïve as compared to treatment experienced [5].

The main quality of the present study is that it is reasonably representative of the real life experience with triple therapy in Italy. Firstly, the proportion of academic and not- academic centers in this study reflects the results of a recent survey of the Italian association for Liver Study (AISF) on centers working on the field of Liver Diseases in Italy where, of the about 200 centers censored, 30% were academic while the remaining were not. Moreover, because the centers who took part in this study were recruited in North, Centre and South Italy and either regions whose administration allowed triple treatment only in patients with advanced diseases, or regions who allowed treatment irrespective of disease severity were included. Although the sample size of our study is not large, we can provide virological results of the entire treatment and follow up for each patient.

With the approach preferred by Italian physicians, high rate of patients with advanced disease, yet did not receive treatment. In order to solve this issue newer IFN-free treatment regimens will ensure higher adherence and treatment suitability in patients with risk of cirrhosis decompensation.

In conclusion, the individualized strategy, adopted by Italian physician in naïve genotype 1 patients, allows larger number of patients to be treated and maximizes responses rates.
